# Multicenter validation of a machine learning model for predicting intrapartum high fever in parturients receiving labor analgesia

**DOI:** 10.3389/fmed.2026.1827290

**Published:** 2026-05-07

**Authors:** Bo Liu, Liang Ling, Chunping Li, Siyan Dou, Jian Zhang, Fei Jia

**Affiliations:** 1 Department of Anesthesiology, Chengdu Jinjiang District Women & Children Health Hospital, Chengdu, Sichuan, China; 2 Department of Anesthesiology, Chongqing General Hospital, Chongqing University, Chongqing, China; 3 Department of Anesthesiology, Sichuan Jinxin Xinan Women & Children’s Hospital, Chengdu, Sichuan, China; 4 Department of Anesthesiology, Sichuan Women's and Children's Hospital/Women's and Children's Hospital, Chengdu Medical College, Chengdu, Sichuan, China

**Keywords:** intrapartum high fever, labor analgesia, machine learning, predictive model, SHAP

## Abstract

**Background:**

Intrapartum high fever (≥38.5 °C) is associated with adverse outcomes, but predicting its occurrence in parturients with intrapartum fever remains difficult. We developed a machine learning model to assess this risk.

**Methods:**

This multicenter retrospective study included parturients who received labor analgesia and developed intrapartum fever (≥38.0 °C) from three Chinese hospitals. The derivation cohort comprised parturients from two hospitals, with parturients from the third hospital serving as an independent external validation cohort. Candidate variables were extracted from electronic health records (EHR). Least absolute shrinkage and selection operator (LASSO) regression was used for feature selection. An extreme gradient boosting (XGBoost) model was developed with hyperparameters optimized via five-fold cross-validation and random search. Model performance was evaluated using area under the receiver operating characteristic curve (AUROC), area under the precision-recall curve (AUPRC), balanced accuracy, and F1-score. The SHapley Additive ExPlanations (SHAP) method was applied to interpret the model.

**Findings:**

A total of 747 parturients were included in this study, of which 238 cases (31.9%) developed intrapartum high fever. Using LASSO regression, six predictors were retained: body mass index (BMI), meconium-stained amniotic fluid, hypertension, anemia, monocyte/lymphocyte ratio (MLR), and platelet/lymphocyte ratio (PLR). The XGBoost model achieved an area under the curve (AUC) of 0.771 in the training set, 0.716 in the test set, and 0.674 in the external validation set. SHAP analysis indicated that BMI was the most important predictive factor.

**Conclusion:**

The XGBoost model demonstrated good performance in predicting intrapartum high fever in parturients with intrapartum fever receiving labor analgesia. SHAP analysis further revealed that BMI was the most important predictive factor in the model.

## 1 Introduction

Intrapartum fever related to labor analgesia is defined as a rise in body temperature (≥38.0 °C) in a labouring woman that occurs after receiving neuraxial analgesia (1). One-third of parturients receiving labor analgesia may face intrapartum fever (2). Intrapartum fever is associated with adverse maternal and neonatal outcomes, such as the need for cesarean delivery, antibiotic use, low Apgar scores in newborns, cardiopulmonary resuscitation, assisted ventilation, and neonatal seizures, and it may even hinder the promotion of labor analgesia in medical institutions (3–8). A more pronounced rise in temperature during intrapartum fever is associated with worse maternal and neonatal outcomes (3, 9). Accurately predicting this risk facilitates risk stratification and resource optimization.

Previous prediction models primarily focused on assessing the risk of intrapartum fever in parturients, providing useful information for identifying those at high-risk of intrapartum fever (10, 11). However, these models did not predict whether fever in these parturients would progress to more severe fever. Additionally, previous prediction models regarding intrapartum fever mainly utilized traditional logistic regression analysis.

In recent years, machine learning models have made significant progress in the medical field, effectively handling large amounts of clinical data and addressing various medical issues (12). As a result, machine learning models have been widely applied to assist physicians in disease prediction and decision support (13, 14). However, these models also have some drawbacks, particularly in terms of result interpretation. Due to their lack of transparency, clinical practitioners often find it challenging to understand the basis of the model’s predictions, posing challenges for the clinical application of these models. To address this issue, the SHapley Additive exPlanations (SHAP) method has been applied (15, 16). This method can clearly reveal the specific impact of each input variable on the outcome, making the model’s predictions more transparent and easier to understand.

## 2 Methods

### 2.1 Study design

This was a multicentre retrospective cohort study. The three participating hospitals were: Chengdu Jinjiang District Women & Children Health Hospital, Sichuan Jinxin Xinan Women & Children’s Hospital, and Sichuan Women’s and Children’s Hospital, all in Chengdu, China. They were selected because (1) they are large tertiary centres with high delivery volumes and established labour analgesia services, and (2) they have standardised electronic health record systems ensuring complete data capture.

A purposive sampling strategy was used for two reasons: (1) to ensure all enrolled parturients had complete key variables required for model development, and (2) to minimise confounding from incomplete outcome data, thereby strengthening internal validity.

The study was approved by the ethics committees of the three hospitals (approval numbers: 2024(20), 2024(2), and 20,230,807-216). Written informed consent was waived due to the retrospective design. The study followed the “Guidelines for Developing and Reporting Machine Learning Predictive Models in Biomedical Research” (17).

### 2.2 Study population

The study population comprised participants aged ≥18 years with a singleton pregnancy who received labour analgesia and developed intrapartum fever (defined as a rise in body temperature ≥38.0 °C after neuraxial analgesia initiation) in the three study hospitals (Chengdu Jinjiang District Women & Children Health Hospital, Sichuan Jinxin Xinan Women & Children’s Hospital, and Sichuan Women’s and Children’s Hospital) in Chengdu, China, during the period from January 1, 2020 to December 31, 2023, with records held in electronic health records (EHR) systems in the study hospitals. Exclusion criteria were: (1) gestational age <37 weeks or >42 weeks; (2) any missing data in the collected variables; and (3) body temperature ≥37.5 °C prior to labour analgesia administration.

### 2.3 Data collection

Data were obtained from the EHR systems of the three study hospitals. The collected variables were categorised as follows: (1) sociodemographic and obstetric characteristics – age, body mass index (BMI), parity, gestational age, premature rupture of membranes (PROM), meconium-stained amniotic fluid, and neonatal weight; (2) maternal medical conditions – gestational diabetes mellitus (GDM), hypertension, anaemia, hepatitis B, and hypothyroidism; (3) laboratory parameters measured immediately after the diagnosis of intrapartum fever (≥38.0 °C) – white blood cell count (WBC), neutrophil count (NEUT), lymphocyte count (Lym), monocyte count (Mono), haemoglobin (Hb), neutrophil-to-lymphocyte ratio (NLR), monocyte-to-lymphocyte ratio (MLR), platelet-to-lymphocyte ratio (PLR), monocyte-to-WBC ratio (Mono/WBC), lymphocyte-to-WBC ratio (Lym/WBC), and C-reactive protein (CRP); and (4) other clinical variables – maximum body temperature during delivery.

### 2.4 Data processing

**Figure 1:**
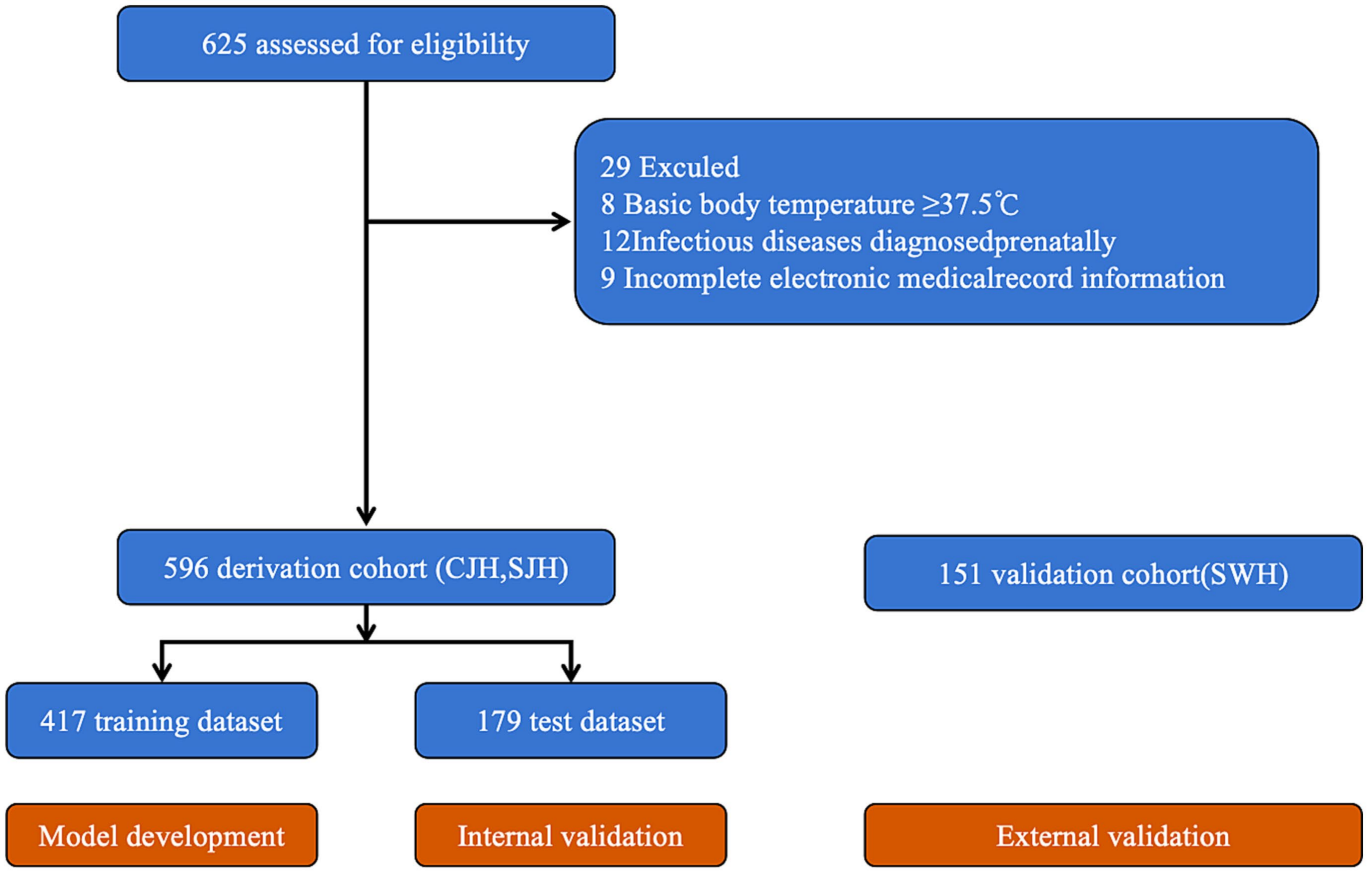
Study flowchart. CJH, Chengdu Jinjiang District Women & Children Health Hospital; SJH, Sichuan Jinxin Xinan Women & Children’s Hospital; SWH, Sichuan Women’s and Children’s Hospital.

Because participants with any missing data were excluded during screening (see Figure 1), no imputation of missing data was necessary. We do not assume missing completely at random (MCAR). The low missing proportion (1.5%) makes complete-case analysis acceptable for model development. To maintain consistency and facilitate model interpretation, continuous variables were standardized using Z-score normalization, and categorical variables were transformed into binary indicators through one-hot encoding to avoid creating false ordinal relationships.

### 2.5 Primary outcome

The primary outcome was the occurrence of intrapartum high fever (T ≥ 38.5 °C). This was defined as the period from the initiation of labor analgesia to 2 h after the delivery of the fetus. During this time, if parturients experienced intrapartum fever and their temperature continued to rise above 38.5 °C, it was recorded as an outcome. All data were confirmed through nursing records, which included maternal temperatures and the associated times.

### 2.6 Model development

We chose the XGBoost algorithm primarily for its excellent performance in binary classification and its ability to effectively prevent overfitting through regularization techniques (18).

The derivation cohort was randomly divided into a training dataset and a test dataset at a 7:3 ratio (19). Within the training dataset, the least absolute shrinkage and selection operator (LASSO) algorithm was employed to identify candidate predictors, which is advantageous as it reduces the number of variables, enhances model interpretability by performing variable selection, and also helps in preventing overfitting by enforcing sparsity in the model (20).

The hyperparameters for the XGBoost algorithm were established through five-fold cross-validation and a random search. This process included various learning rates (0.01, 0.03, 0.05, and 0.07), weights for positive samples (1, 10, 20, and 30), maximum depths for the decision trees (3, 4, and 5), numbers of decision trees (50, 75, and 100), subsample ratios for training instances (0.8 and 1), gamma values (0.5, 0.7, and 0.9), and subsample ratios for columns (0.8 and 1) (15).

### 2.7 Model validation

Model performance was evaluated using AUROC, AUPRC, balanced accuracy, and F1-score. The threshold maximizing the sum of sensitivity and specificity was selected for single-threshold metrics (21, 22). Calibration plots were used to assess agreement between predicted and observed risks. Model interpretability was enhanced using SHAP values to illustrate feature impacts (23, 24).

### 2.8 Sample size

This prediction model incorporates 23 predictors, each requiring a minimum of 10 participants (13). Therefore, we need at least 230 participants. Considering a 10% missing data rate, the total number of participants should at least 256 participants. Our derivation cohort included 596 participants, thereby meeting the aforementioned criteria.

### 2.9 Statistical analysis

The Kolmogorov–Smirnov test was used to evaluate the normal distribution of continuous variables. For normally distributed continuous variables, data are presented as the mean ± standard deviation (SD) and were analyzed via the independent-sample *t*-test. Nonnormally distributed continuous variables are expressed as the median (M) and interquartile range (IQR) and were compared using the Mann–Whitney U-test. Categorical variables are described as percentages (%), with group comparisons conducted using the chi-square test. A *p*-value of less than 0.05 was considered statistically significant. The model development and validation were performed using R version 4.3.1.

## 3 Results

### 3.1 Parturients characteristics

A total of 747 parturients were included in this study, of which 238 cases (31.9%) developed intrapartum high fever (Figure 1). Within the derivation cohort of 596 participants, 197 (33.1%) experienced intrapartum high fever, while in the external validation cohort of 151 participants, 41 (27.2%) developed the outcome. The characteristics of the training dataset within the derivation cohort are presented in Table 1. The characteristics of the test dataset from the derivation cohort and the external validation cohort are presented in Supplementary Tables S1, S2, respectively.

**Table 1:** A comparison of characteristics in the training dataset of the derivation cohort.

Characteristics	Overall	Intrapartum high fever		*p*
	*n* = 417	Yes (130)	No (287)	
Parturient characteristics				
Age (years)	28.0(27–31)	29.0(27–31)	28.0(27–31)	0.595
BMI (kg/m^2^)	24.9 ± 3.7	25.5 ± 3.8	24.6 ± 3.6	0.016
Gestational age (w)	39.9(39.0–40.3)	39.9(38.9–40.4)	39.9(39.0–40.4)	0.723
Meconium-stained amniotic fluid (%)	115(27.6)	41(31.5)	74(25.8)	0.238
Primiparity (%)	399(95.7)	124(95.4)	275(95.8)	0.800
PROM (%)	140(33.6)	45(34.6)	95(33.1)	0.823
Macrosomia (%)	29(7.0)	10(7.7)	19(6.6)	0.682
Comorbidity				
GDM (%)	103(24.7)	32(24.6)	71(24.7)	1.000
Hypertension (%)	28(6.7)	12(9.2)	16(5.6)	0.204
Anemia (%)	64(15.3)	27 (20.8)	37 (12.9)	0.056
Hepatitis B (%)	17(4.1)	6(4.6)	11(3.8)	0.790
Hypothyroidism (%)	47(11.3)	13(10.0)	34(11.8)	0.621
Laboratory tests in intrapartum fever				
WBC count (10^9^/L)	15.1(12.9–17.2)	15.0(13.0–17.0)	15.2(12.9–17.4)	0.579
NEUT count (10^9^/L)	13.1(11.0–15.2)	13.2(11.2–15.1)	13.1(11.0–15.4)	0.747
LYM count (10^9^/L)	1.0(0.8–1.3)	1.0(0.8–1.3)	1.0(0.8–1.3)	0.820
CRP(mg/L)	18.3(10.1–33.2)	18.7(10.9–32.6)	17.5(10.0–33.4)	0.425
NLR (%)	12.8(10.0–16.5)	12.8(9.9–17.3)	12.7(10.1–16.4)	0.992
MLR (%)	0.8(0.6–1.0)	0.8(0.6–0.9)	0.8(0.6–1.0)	0.079
PLR (%)	156.9(116.5–201.3)	163.7(118.3–212.1)	153.1(116.0–194.6)	0.088
Mono/WBC	0.05(0.04–0.06)	0.05(0.04–0.06)	0.05(0.05–0.06)	0.109
Lym/WBC	0.07(0.05–0.09)	0.07(0.05–0.09)	0.07(0.05–0.08)	0.806
NEUT/WBC	0.87(0.85–0.90)	0.88(0.85–0.90)	0.87(0.85–0.90)	0.708

BMI, body mass index; GDM, gestational diabetes; PROM, premature rupture of membranes; WBC, white blood cell; NEUT, neutrophil; Lym, lymphocyte; NLR, neutrophil/lymphocyte; MLR, monocyte/lymphocyte; PLR, platelet/lymphocyte; Mono, monocyte.

### 3.2 Predictive factor

**Figure 2:**
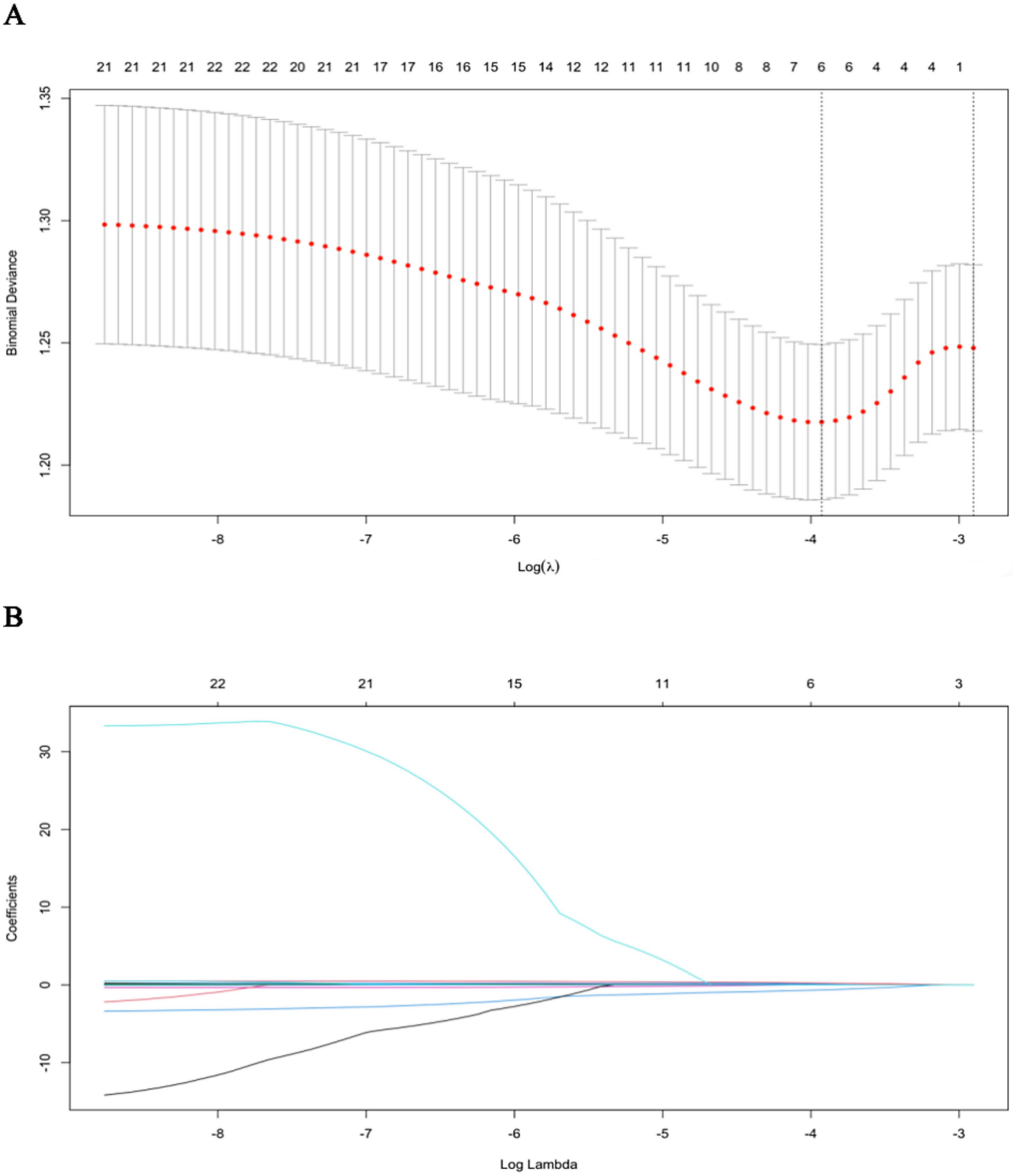
LASSO regression analysis results. **(A)** Cross-validated binomial deviance plotted against the log of the regularization parameter (*λ*). The red dotted line indicates the λ value selected as `lambda min`, which corresponds to the lowest binomial deviance. The gray bars represent the standard error at each λ level. **(B)** Coefficient paths for the 23 features related to fever during labor, as λ increases. Each line represents the coefficients of a feature, with important variables being retained while others are shrunk toward zero.

LASSO regression shrinks variable coefficients through a penalty function and achieves variable selection, with the penalty controlled by the hyperparameter *λ*. Through ten-fold cross-validation, we determined the optimal λ value by selecting lambda min, which minimizes the binomial deviance (Figure 2A). Figure 2B illustrates the LASSO coefficient paths for 23 features related to intrapartum high fever during labor; as the penalty increases, the selection of important variables is enhanced. Ultimately, we identified six predictive factors: BMI, meconium-stained amniotic fluid, hypertension, anemia, MLR, and PLR.

### 3.3 Building machine learning models

**Figure 3:**
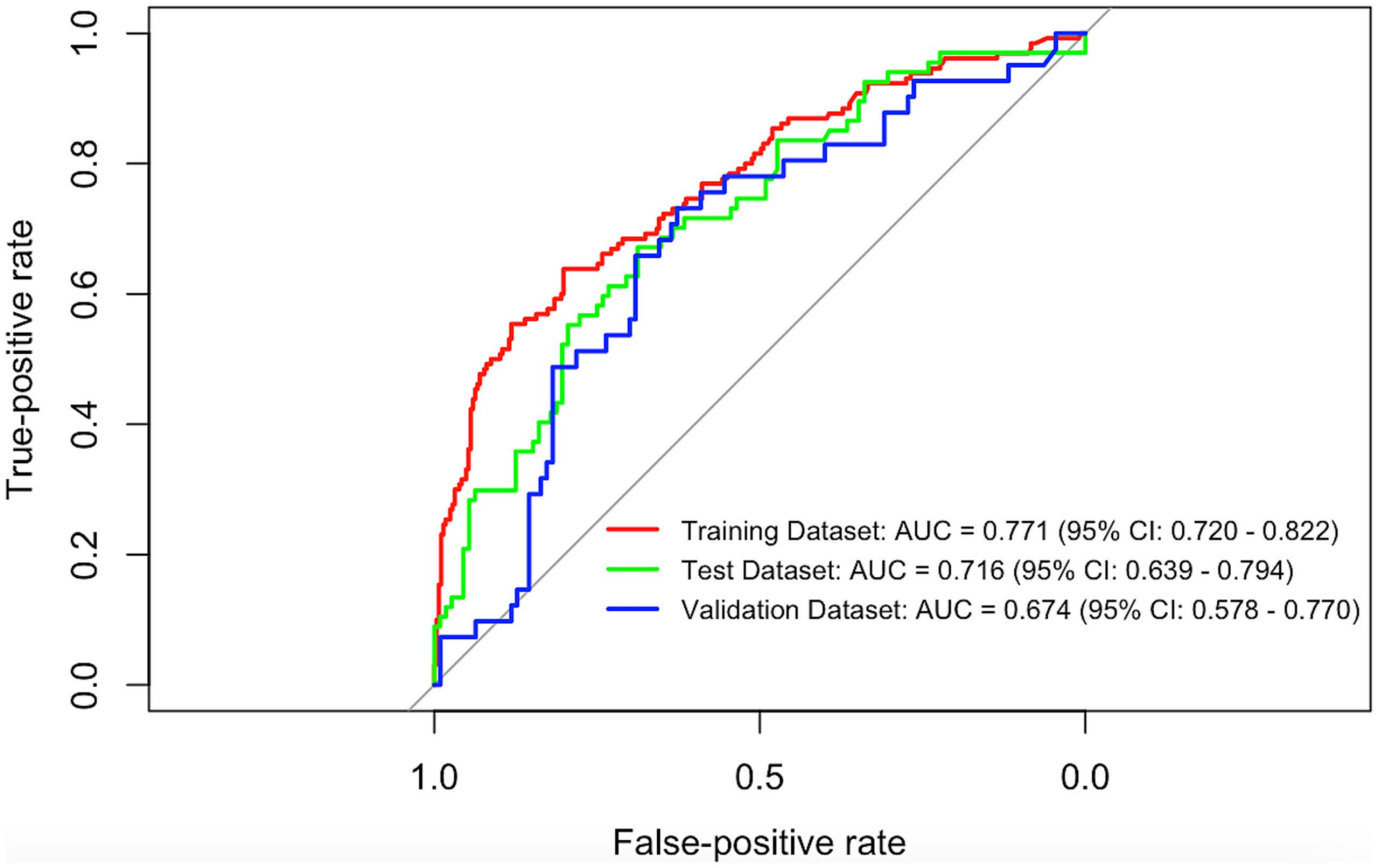
AUROC curves for the XGBoost model in the derivation and validation datasets.

The XGBoost model achieved AUROCs of 0.771 (95% CI, 0.720–0.822) in the training dataset, 0.716 (95% CI, 0.639–0.794) in the test dataset, and 0.674 (95% CI, 0.578–0.770) in the external validation dataset (Figure 3). The AUPRCs were 0.659, 0.614, and 0.391, respectively; balanced accuracies were 0.805, 0.824, and 0.488; and F1 scores were 0.228, 0.164, and 0.140 (Table 2). Calibration curves for all datasets after recalibration are presented in Supplementary Figure S1.

**Table 2:** Model evaluation results on different datasets.

Characteristics	AUROC	AUPRC	Sensitivity	Specificity	Precision	Balanced accuracy	F1 score
Training dataset	0.771 (0.720–0.822)	0.659	0.895	0.716	0.131	0.805	0.228
Testing dataset	0.716 (0.639–0.794)	0.614	1.000	0.647	0.090	0.824	0.164
Validation dataset	0.674 (0.578–0.770)	0.391	0.250	0.726	0.098	0.488	0.140

AUROC, area under the receiver operating characteristic curve; AUPRC, area under the precision-recall curve.

### 3.4 SHAP analysis

**Figure 4:**
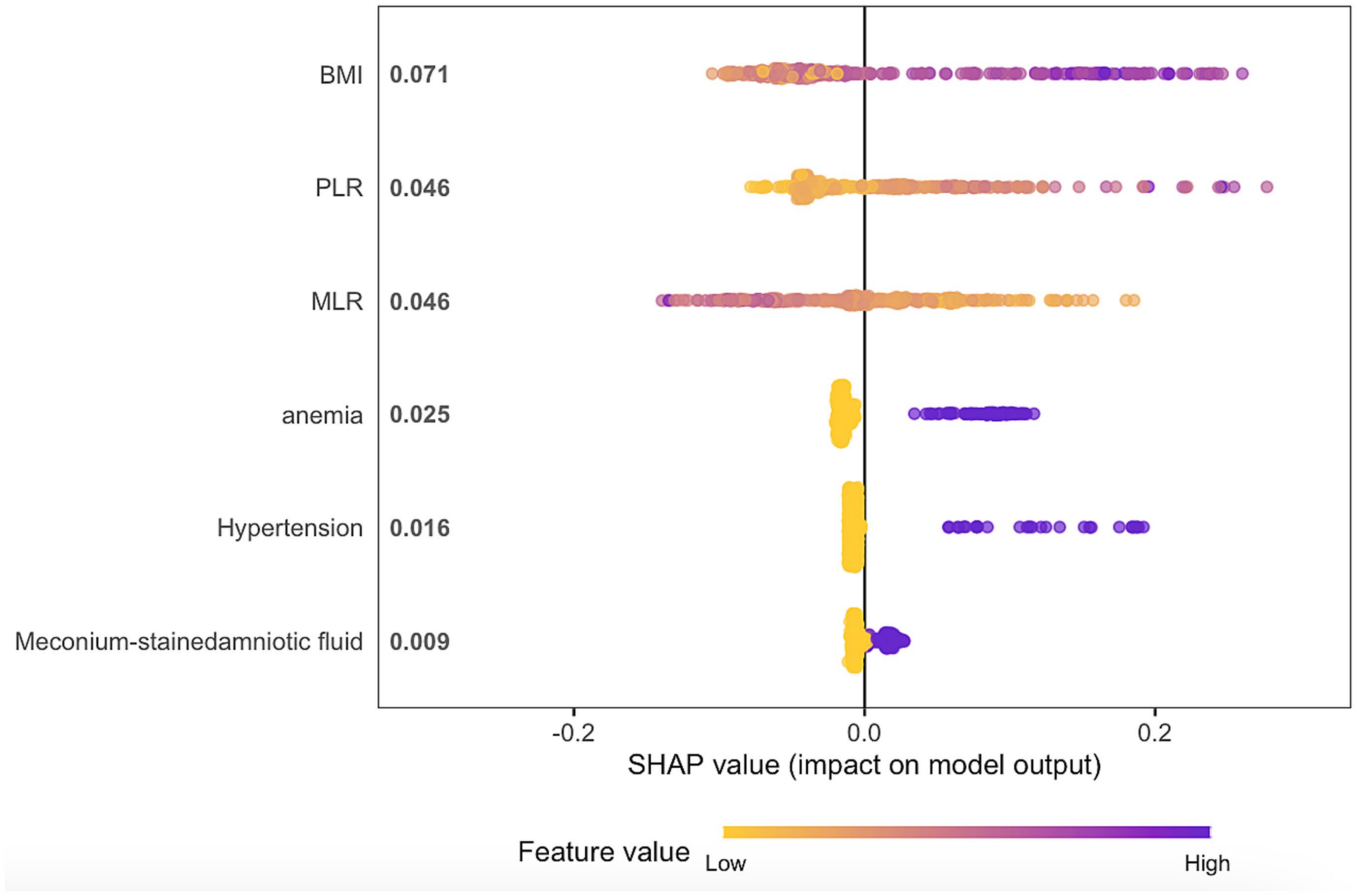
The SHAP summary plot illustrates the six features of the final model. Purple and yellow indicate larger and smaller values of the features, respectively, with purple dots representing high feature values and yellow dots indicating the opposite. Large SHAP values denote a significant impact of individual features on the outcome. In this figure, higher BMI, higher PLR, anemia, hypertension, meconium-stained amniotic fluid, and lower MLR are associated with an increased risk of intrapartum high fever.

Based on the SHAP summary plot (Figure 4), BMI was identified as the most influential predictor for intrapartum high fever, followed by PLR, MLR, anemia, hypertension, and meconium-stained amniotic fluid.

## 4 Discussion

We developed and externally validated a machine learning model to predict the risk of intrapartum high fever following intrapartum fever in parturients receiving labor analgesia. Utilizing 23 easily extractable EHR variables, the model reduces reliance on clinicians’ subjective judgments and demonstrates good discrimination ability in predicting intrapartum high fever. Although performance slightly decreased during external validation, it remained within an acceptable range. SHAP analysis revealed the specific impact of each feature on the risk of intrapartum high fever, offering an in-depth understanding of the model’s predictive basis.

A key advantage of our machine learning model is its reliance on just 23 easily extractable EHR variables, minimizing subjective judgment in risk assessment and reducing biases for real-time automated risk calculations (25–27). Traditional predictive models often use numerous variables, which can limit their clinical applicability; for instance, one study relied on hundreds of features to predict peripheral artery disease, affecting generalizability and accuracy due to missing data (28). Our research, using LASSO regression, identified six key predictive factors, significantly streamlining input variables. This improvement enhances our model’s applicability across various clinical settings, providing clinicians with a straightforward and reliable tool for predicting intrapartum high fever risks during labor analgesia.

External validation is essential for the credibility and clinical applicability of machine learning models, as it assesses their generalizability across different populations and settings (29). In our study, we not only allocated a test dataset but also conducted independent external validation at another hospital. We noted a slight decline in model performance, likely due to differences in parturient characteristics and hospital environments. Future research should explore how these variables influence external validation outcomes, focusing on the model’s applicability and reliability in various clinical contexts. This will help refine the model and enhance its value in diverse clinical settings.

One of the main advantages of SHAP plots is their ability to quantify the contribution of each feature to model predictions, helping clinicians understand the rationale behind the model (30). This interpretability enhances transparency in complex machine learning models and identifies key factors for risk prediction, thus boosting credibility (31). The SHAP summary plot indicates that BMI is the most significant factor influencing risk, followed by PLR, MLR, and anemia, while meconium-stained amniotic fluid and hypertension have lesser impacts. By improving transparency, these explainable techniques facilitate the integration of machine learning into clinical practice. However, further prospective studies are needed to validate the reliability of these predictions in real-world settings.

The final selected variables in our model align with previous studies on intrapartum fever risk factors, including higher BMI, meconium-stained amniotic fluid, anemia, MLR, PLR, and hypertension (13, 14, 32). Notably, higher BMI emerged as the most significant predictor of intrapartum high fever. This may be due to higher body fat levels, which secrete inflammatory cytokines and promote systemic inflammation (33, 34). Additionally, obesity can impair immune responses, leading to more severe inflammatory reactions during infections (35). Metabolic issues like insulin resistance may also increase susceptibility to abnormal inflammatory responses (36). Furthermore, elevated PLR during fever underscores the role of inflammation, and hypertension has been associated with high fever (37).

In our final model, only MLR and PLR were identified as risk factors that could be assessed after the onset of intrapartum fever, while the other risk factors could be evaluated beforehand. Although previous studies have reported primiparity and PROM as risk factors for intrapartum fever, these were not replicated in our model predicting intrapartum high fever progression (13, 14). This discrepancy may stem from differences in study outcomes and populations; earlier studies focused on the occurrence of intrapartum fever in participants who had not yet experienced it, whereas our study examined high fever in parturients who had already developed intrapartum fever.

Calibration is crucial in evaluating clinical prediction models for comparing predicted event rates with actual outcomes (38). Our study showed a slight decline in calibration during external validation, influenced by factors like event occurrence rates, predictor variable distribution, missing data, and treatment strategies for intrapartum fever (39). Although the model exhibited good predictive ability in both internal and external validation, these calibration errors underscore the need for performance assessment in real clinical settings, as variations in parturient characteristics across hospitals can impact prediction accuracy. Future research should focus on these factors to enhance the model’s applicability and reliability. Further validation aims to optimize the model’s practical value in clinical decision-making, ultimately benefiting parturients and their newborns.

Our model serves as a screening tool to identify parturients needing additional monitoring and intervention. Although it showed decreased accuracy and sensitivity during external validation, it is essential for recognizing high-risk individuals and preventing severe outcomes. Continuous monitoring of temperature and clinical indicators can help promptly detect intrapartum high fever risks. The model also facilitates informed consent and encourages parturient involvement in care, while its interpretability aids in understanding risk factors and developing personalized care plans. Despite its limitations, we believe this model can enhance the management of intrapartum high fever and improve maternal and neonatal health outcomes.

This study has several limitations. First, the retrospective design may introduce selection bias and information bias. Selection bias could arise because we excluded parturients with missing key variables, potentially creating a study sample that differs from the full target population. Information bias might stem from inaccuracies or inconsistencies in electronic health records, such as incomplete documentation of body temperature or laboratory tests, as these data were not originally collected for research purposes. Second, the study population was restricted to parturients receiving labour analgesia, limiting the generalisability of the model to those using other analgesia methods or no analgesia. Third, the relatively small sample size and the single-city (Chengdu) geographic scope may limit external validity. Fourth, we could not incorporate known inflammatory biomarkers (e.g., interleukins) that may be associated with intrapartum fever (40). Additionally, we did not compare the full-feature XGBoost model with the LASSO-selected model, and LASSO as a linear method may have excluded some important nonlinear predictors. Finally, despite collecting a wide range of clinical variables, some potential confounders might have been omitted.

To address these limitations, prospective multicentre studies with larger sample sizes and diverse populations are needed to validate the model. Future studies should also include standardised collection of inflammatory markers and explore the model’s performance in parturients without labour analgesia. Additionally, prospective designs with predefined data collection protocols would help minimise selection and information bias.

## 5 Conclusion

In summary, we developed and externally validated an explainable machine learning model that utilized easily extractable EHR variables to predict the occurrence of intrapartum high fever following intrapartum fever in parturients receiving labor analgesia. Future prospective studies are essential to further validate the model’s performance and explore its clinical utility in optimizing the management of intrapartum fever, ultimately improving maternal and neonatal health outcomes.

## Data Availability

The raw data supporting the conclusions of this article will be made available by the authors, without undue reservation.
