# Supplementary material for: Multicenter validation of a machine learning model for predicting intrapartum high fever in parturients receiving labor analgesia
Source: Front Med (Lausanne). 2026 May 7;13:1827290. doi: 10.3389/fmed.2026.1827290 (PMC13190175; doi:10.3389/fmed.2026.1827290)
Supplement: Supplementary file 1 [file Data_Sheet_1.PDF]

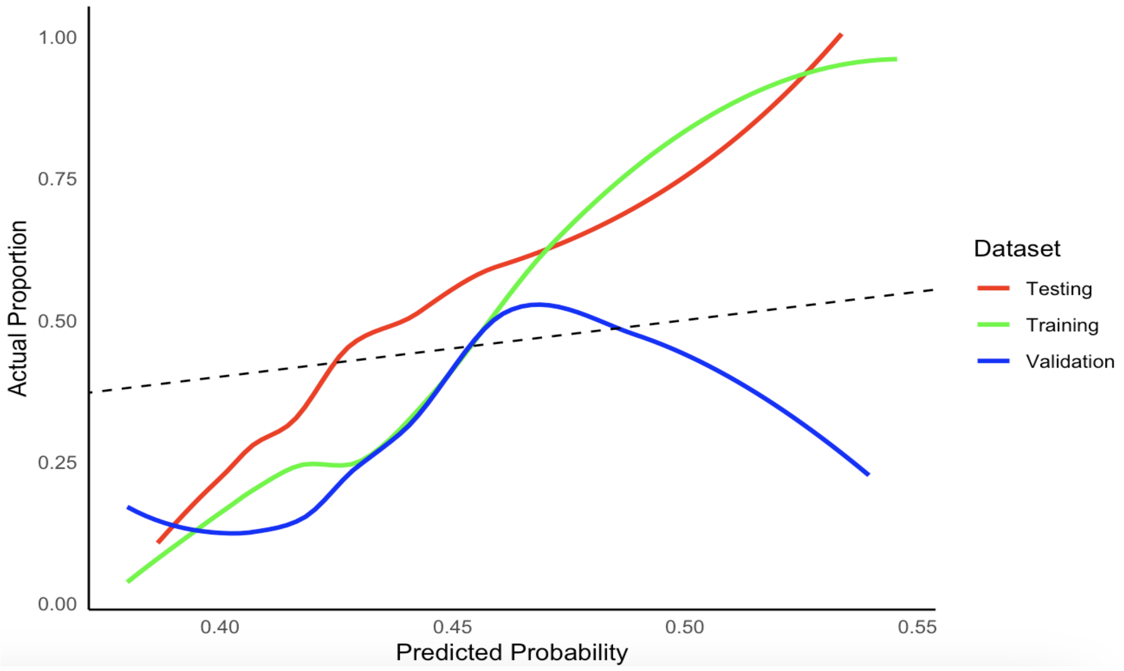

Figure S1.Calibration Curve: The calibration curve illustrates the relationship between the predicted probabilities of the model and the actual observed outcomes across different datasets. The diagonal dashed line indicates the ideal calibration scenario, where the predicted probabilities closely align with the observed probabilities. The degree of deviation from this line reflects the accuracy and calibration effectiveness of the model across the various datasets.
