# Supplementary material for: Multicenter validation of a machine learning model for predicting intrapartum high fever in parturients receiving labor analgesia
Source: Front Med (Lausanne). 2026 May 7;13:1827290. doi: 10.3389/fmed.2026.1827290 (PMC13190175; doi:10.3389/fmed.2026.1827290)
Supplement: Supplementary file 2 [file Table_1.DOCX]

**Table S1. presents a comparison of characteristics in the test dataset of the derivation cohort.**

| **Characteristics** | Intrapartum high fever | | ***P* value** |
| --- | --- | --- | --- |
|  | **Yes（67）** | **No（112）** |  |
| **Parturient characteristics** |  | | |
| Age (years) | 28.0(27.0-31.0) | 29.0(27.0-31.0) | 0.761 |
| BMI (kg/m^2^) | 26.3 ± 3.1 | 23.8 ± 3.8 | ＜0.001 |
| Gestational age (w) | 39.7(39.0-40.4) | 40.0(39.3-40.4) | 0.477 |
| Meconium-stained amniotic fluid (%) | 24(35.8) | 18(16.1) | 0.003 |
| Primiparity (%) | 62(92.5) | 106(94.6) | 0.749 |
| PROM (%) | 21(31.3) | 33(29.5) | 0.867 |
| Macrosomia(%) | 5(7.5) | 9(8.0) | 1.000 |
| **Comorbidity** |  |  |  |
| GDM(%) | 16(23.9) | 24(21.4) | 0.714 |
| Hypertension(%) | 4(6.0) | 2(1.8) | 0.199 |
| Anemia(%) | 10(14.9) | 13(11.6) | 0.645 |
| Hepatitis B (%) | 4(6.0) | 9(8.0) | 0.769 |
| Hypothyroidism (%) | 5(7.5) | 20(17.9) | 0.073 |
| **Laboratory tests in Intrapartum Fever** |  |  |  |
| WBC count (10^9^/L) | 15.4(12.8-18.4) | 15.1(13.3-17.5) | 0.815 |
| NEUT count (10^9^/L) | 13.7(10.6-16.0) | 13.1(11.5-15.4) | 0.913 |
| LYM count (10^9^/L) | 1.1(0.8-1.5) | 1.1(0.8-1.2) | 0.321 |
| CRP(mg/L) | 16.2(10.4-35.7) | 18.3(9.3-34.1) | 0.717 |
| NLR (%) | 12.0(8.7-16.9) | 12.3(10.0-16.0) | 0.558 |
| MLR (%) | 0.8(0.5-0.9) | 0.8(0.6-1.0) | 0.687 |
| PLR (%) | 153.1(109.1-214.6) | 153.9(115.7-197.0) | 0.986 |
| Mono/WBC | 0.06(0.05-0.06) | 0.05(0.04-0.06) | 0.382 |
| Lym/WBC | 0.07(0.05-0.10) | 0.07(0.06-0.09) | 0.615 |
| NEUT/WBC | 0.87(0.84-0.90) | 0.88(0.85-0.89) | 0.431 |

Abbreviations: BMI body mass index, GDM gestational diabetes, PROM premature rupture of membranes,WBC white blood cell, NEUT neutrophil, Lym lymphocyte, NLR Neutrophil/

Lymphocyte, MLR Monocyte/Lymphocyte, PLR Platelet/Lymphocyte, Mono monocyte.
