# Supplementary material for: Multicenter validation of a machine learning model for predicting intrapartum high fever in parturients receiving labor analgesia
Source: Front Med (Lausanne). 2026 May 7;13:1827290. doi: 10.3389/fmed.2026.1827290 (PMC13190175; doi:10.3389/fmed.2026.1827290)
Supplement: Supplementary file 3 [file Table_2.DOCX]

**Table S2 presents a comparison of characteristics in the validation cohort.**

| **Characteristics** | Intrapartum high fever | | ***P* value** |
| --- | --- | --- | --- |
|  | **Yes（41）** | **No（110）** |  |
| **Parturient characteristics** |  | | |
| Age (years) | 30.0(27.0-33.0) | 29.0(27.0-32.0) | 0.726 |
| BMI (kg/m^2^) | 27.1 ± 3.1 | 26.3 ± 2.9 | 0.110 |
| Gestational age (w) | 39.6(38.6-40.3) | 39.4(38.6-40.1) | 0.725 |
| Meconium-stained amniotic fluid (%) | 11(26.8) | 11(10.0) | **0.017** |
| Primiparity (%) | 37(90.2) | 108(98.2) | **0.047** |
| PROM (%) | 18(43.9) | 47(42.7) | 1.000 |
| Macrosomia(%) | 0(0.00) | 1(0.01) | 1.000 |
| **Comorbidity** |  |  |  |
| GDM(%) | 13(31.7) | 25(22.7) | 0.294 |
| Hypertension(%) | 14(34.1) | 7(6.4) | **＜0.001** |
| Anemia(%) | 14(34.1) | 24(21.8) | 0.142 |
| Hepatitis B (%) | 3(7.3) | 8(7.3) | 1.000 |
| Hypothyroidism (%) | 3(7.3) | 18(16.4) | 0.192 |
| **Laboratory tests in Intrapartum Fever** |  |  |  |
| WBC count (10^9^/L) | 14.4(12.1-16.1) | 14.2(12.2-16.3) | 0.908 |
| NEUT count (10^9^/L) | 11.9± 3.3 | 12.1 ± 3.2 | 0.717 |
| LYM count (10^9^/L) | 1.1(0.8-1.6) | 1.2(0.9-1.4) | 0.957 |
| CRP(mg/L) | 12.9(8.6-26.6) | 10.3(4.9-17.0) | 0.015 |
| NLR (%) | 10.3(6.9-14.4) | 10.0(7.3-13.8) | 0.897 |
| MLR (%) | 0.7(0.5-0.9) | 0.7(0.5-0.9) | 0.357 |
| PLR (%) | 134.4(110.4-167.5) | 128.4(96.9-178.5) | 0.732 |
| Mono/WBC | 0.06(0.05-0.06) | 0.06(0.05-0.07) | 0.109 |
| Lym/WBC | 0.09 ± 0.03 | 0.09 ± 0.03 | 0.948 |
| NEUT/WBC | 0.85(0.81-0.88) | 0.85(0.82-0.88) | 0.927 |

Abbreviations: BMI body mass index, GDM gestational diabetes, PROM premature rupture of membranes,WBC white blood cell, NEUT neutrophil, Lym lymphocyte, NLR Neutrophil/

Lymphocyte, MLR Monocyte/Lymphocyte, PLR Platelet/Lymphocyte, Mono monocyte.
